# Association of Sialic Acid–Binding Immunoglobulin-Like Lectin 15 With Phenotypes in Esophageal Squamous Cell Carcinoma in the Setting of Neoadjuvant Chemoradiotherapy

**DOI:** 10.1001/jamanetworkopen.2022.50965

**Published:** 2023-01-17

**Authors:** Sha Zhou, Yuting Wang, Rui Zhang, Weian Zeng, Shiliang Liu, Songran Liu, Mengzhong Liu, Hong Yang, Mian Xi

**Affiliations:** 1 State Key Laboratory of Oncology in South China, Collaborative Innovation Centre for Cancer Medicine, Guangdong Esophageal Cancer Institute, Guangzhou, China; 2 Department of Anesthesiology, Sun Yat-sen University Cancer Center, Guangzhou, China; 3 Department of Radiation Oncology, Sun Yat-sen University Cancer Center, Guangzhou, China; 4 Department of Radiotherapy, The First Affiliated Hospital of Kunming Medical University, Kunming, China; 5 School of Pharmaceutical Sciences and Yunnan Key Laboratory of Pharmacology for Natural Products, Kunming Medical University, Kunming, China; 6 Department of Pathology, Sun Yat-sen University Cancer Center, Guangzhou, China; 7 Department of Thoracic Surgery, Sun Yat-sen University Cancer Center, Guangzhou, China

## Abstract

**Importance:**

Sialic acid–binding immunoglobulin-like lectin 15 (Siglec-15) is a novel immune checkpoint molecule that is highly homologous to programmed cell death ligand 1 (PD-L1), but information remains limited about its role in esophageal squamous cell carcinoma (ESCC).

**Objective:**

To explore the expression pattern and association of Siglec-15 with outcomes among patients with ESCC who received neoadjuvant chemoradiotherapy (CRT).

**Design, Setting, and Participants:**

This retrospective cohort study was conducted at an academic institution in China. Participants included patients with ESCC who underwent neoadjuvant CRT and esophagectomy between June 2002 and December 2018. Multiplexed immunofluorescence staining was used to evaluate the expression of Siglec-15 and PD-L1 in tumor cells (TCs) or tumor-associated macrophages based on pre-CRT biopsies. Different immune phenotypes have been proposed and further validated in an independent cohort. Data analysis was conducted from January to May 2021.

**Exposures:**

Siglec-15 or PD-L1 positivity vs negativity.

**Main Outcomes and Measures:**

Pathologic complete response (pCR), overall survival (OS), and recurrence-free survival (RFS).

**Results:**

Of 130 participants (median [range] age, 56 [42-73] years; 108 [83.1%] male participants) in the primary cohort, 58 patients (44.6%) achieved a pCR after neoadjuvant CRT. Siglec-15 and PD-L1 were detected in both TCs and macrophages. The percentage of Siglec-15–positive macrophages was notably higher than that of Siglec-15–positive TCs (median [IQR]: 34.4% [12.7%-64.3%] vs 4.8% [0.7%-25.6%]; *P* < .001). TC–Siglec-15 expression was significantly and positively associated with macrophage–Siglec-15 expression (*r* = 0.78; *P* < .001). Siglec-15 positivity was significantly associated with a higher rate of pCR (37 of 70 [52.9%] vs 21 of 60 [35.0%]; *P* = .04), more favorable OS (hazard ratio [HR], 0.46; 95% CI, 0.25-0.85; *P* = .01), and RFS (HR, 0.48; 95% CI, 0.26–0.88; *P* = .02). However, PD-L1 positivity in TCs was negatively associated with survival. Stratification analysis further revealed that patients with combined Siglec-15 positivity and PD-L1 negativity had better survival than those with other phenotypes. Major findings were reproducible in a validation cohort with 55 patients.

**Conclusions and Relevance:**

In this cohort study of patients with ESCC receiving neoadjuvant CRT, Siglec-15 positivity was associated with a better pathological response and more favorable survival. Siglec-15 could serve as a novel biomarker to identify potential candidates that may benefit from immunotherapy combined with CRT.

## Key Points

**Question:**

What phenotypes are associated with the novel immune suppressor sialic acid–binding immunoglobulin-like lectin 15 (Siglec-15) in patients with esophageal squamous cell carcinoma who received neoadjuvant chemoradiotherapy and surgery?

**Findings:**

In this cohort study involving 130 patients, Siglec-15 expression in tumor cells was positively associated with its expression in tumor-associated macrophages. Siglec-15 positivity was associated with a higher rate of pathologic complete response and more favorable survival than Siglec-15 negativity.

**Meaning:**

These results suggest that Siglec-15–targeted agents may be an effective strategy to improve pathologic response and survival in patients with esophageal squamous cell carcinoma.

## Teaser

This cohort study explores the expression pattern and association of sialic acid–binding immunoglobulin-like lectin 15 (Siglec-15) with outcomes among patients with esophageal squamous cell carcinoma (ESCC) who received neoadjuvant chemoradiotherapy.

## Introduction

Esophageal cancer (EC) is among the most aggressive malignant neoplasms, accounting for 3.1% of all cancer cases and 5.5% of cancer-related deaths worldwide.^1^ For locally advanced EC, neoadjuvant chemoradiotherapy (CRT) followed by surgery has been recommended as the standard care in recent years.^2,3,4^ After neoadjuvant CRT, approximately 20% to 40% of patients with EC can achieve pathologic complete response (pCR) with significantly favorable survival.^5^ For patients with a poor response to neoadjuvant CRT, clinical outcomes are still far from satisfactory.^6,7,8^ Thus, innovative approaches are urgently needed to improve the pCR rate and survival among patients with EC treated with neoadjuvant CRT.

The emergence of immunotherapy and immune checkpoint inhibitors, especially anti–programmed cell death 1 (PD-1) and programmed cell death ligand 1 (PD-L1), has greatly changed the therapeutic landscape of various malignant neoplasms.^9,10,11^ Given that most patients exhibit de novo or adaptive resistance to PD-1/PD-L1 inhibitor monotherapy, the overall efficacy of PD-1/PD-L1 inhibitors is unsatisfactory.^12^ For example, previous studies have consistently demonstrated that only 17% to 30% of patients with EC respond to PD-1/PD-L1 inhibitors with clinical benefit.^13,14,15,16^ Therefore, exploring novel immunomodulatory targets is necessary and extensive research is ongoing.

Sialic acid–binding immunoglobulin-like lectins (Siglecs) play important roles in recognizing sialylated glycans and in regulating immune homeostasis.^17^ Siglec-15, a highly conserved member of the Siglec family that functions as an osteoclast modulator, was identified as a novel immune suppressor by Chen et al.^18^ Siglec-15 mRNA has limited expression in most normal human tissues, but is upregulated in a variety of human cancer cells and tumor-infiltrating myeloid cells.^18,19^ Despite its high homology with the B7 family members, Siglec-15 is mutually exclusively expressed with PD-L1 in human lung cancer tissues.^18^ A preclinical study demonstrated that Siglec-15 works independently of the PD-1/PD-L1 pathway in immune regulation,^18^ suggesting that blocking Siglec-15 may provide an attractive immune therapeutic strategy for patients who would not benefit from anti–PD-1/PD-L1 therapy.

An increasing number of studies have explored the expression signature and significance of Siglec-15 in diverse cancers.^19,20,21,22^ Li et al^19^ found that Siglec-15 was upregulated in EC but did not exhibit prognostic value based on bioinformatic analysis. However, no studies have investigated the expression pattern of Siglec-15 in tumor cells (TCs) or tumor-associated macrophages in patients with EC. Moreover, the association between Siglec-15 and pathological response to neoadjuvant CRT remains unknown. Thus, this study aimed to evaluate the expression of Siglec-15 in TCs and tumor-associated macrophages and the potential association of different immune phenotypes with pathologic response and survival outcomes in patients with esophageal squamous cell carcinoma (ESCC) who underwent neoadjuvant CRT and esophagectomy.

## Methods

This study was approved by the institutional ethics committees of Sun Yat-sen University Cancer Center, and the requirement for informed consent was waived owing to its retrospective nature. This study followed the Strengthening the Reporting of Observational Studies in Epidemiology (STROBE) reporting guideline.

### Patients

A total of 130 patients with ESCC who received neoadjuvant CRT followed by esophagectomy from a prospectively maintained database at the Sun Yat-sen University Cancer Center from June 2002 to July 2017 were enrolled in this study as the primary cohort. An independent cohort of 55 patients who satisfied the inclusion criteria at our institution between August 2017 and December 2018 was used for validation. The eligibility criteria included clinical stage II to IVa ESCC according to the eighth edition TNM staging system of the American Joint Committee on Cancer, external-beam radiotherapy with concurrent platinum-based chemotherapy followed by Ivor-Lewis esophagectomy with curative intent, and pretreatment biopsy specimens obtained.

All patients received radiotherapy with a total dose of 40.0 to 45.5 Gy using 3-dimensional conformal radiotherapy or intensity-modulated radiotherapy. Esophagectomy was conducted 6 to 8 weeks after the completion of CRT. We defined pCR as the absence of viable cancer cells in all layers of the esophagus and resected lymph nodes. Patients were followed up postoperatively every 3 months for the first 2 years, every 6 months for the next 3 years, and annually thereafter.

### Immunohistochemistry

The types of primary antibodies are detailed in eMethods in Supplement 1. Immunohistochemistry (IHC) staining and analysis were conducted to test the altered protein expression of Siglec-15 in human ESCC specimens. Briefly, formalin-fixed and paraffin-embedded tumor tissue sections were deparaffinized, rehydrated, placed in 0.3% H_2_O_2_ to diminish the activity of endogenous peroxidase, and then heated in a pressure-boiling container for antigen retrieval. The following incubation with anti–Siglec-15 antibody and secondary antibody was performed at 4 °C and room temperature, respectively. The sections were then stained with diaminobenzidine and counterstained with hematoxylin. A Eclipse Advanced Research Microscope was used for image acquisition (Nikon). IHC staining scores were determined by combining the proportion of positively stained tumor cells (graded as 1, <1%; 2, 1%-10%; 3, 10%-50%; and 4, ≥50%) and the intensity of staining (graded as 0, negative staining; 1, weak staining; 2, moderate staining; and 3, strong staining).

### Multiplex Staining and Multispectral Imaging

Multiplexed immunofluorescence staining using formalin-fixed paraffin-embedded tissues cut into 4-μm thick sections was performed following the standard manufacturer’s protocols, as described previously.^23^ Multiplex-stained slides were imaged using the Mantra System (PerkinElmer), which establishes an image cube by capturing the fluorescent spectra at 20-nm wavelength intervals from 420 to 720 nm. The details of imaging methods are provided in eMethods in Supplement 1.

### Image Analysis

Images of single stained and unstained sections were applied to extract the spectrum of each fluorescein and the tissue autofluorescence, respectively, to establish the spectral library, which was required for multispectral unmixing using inForm image analysis software (PerkinElmer) and to obtain reconstructed images of sections with the autofluorescence removed. Cells displaying unequivocal membranous or cytoplasmic Siglec-15 or PD-L1 staining, regardless of intensity, were considered antibody-specific stained cells. The inForm software identified positively stained cells by setting a reasonable threshold to accurately calculate the number of all cells and the number of positive cells. ESCC TCs were identified as membranous pan-cytokeratin (PAN-CK) postitive and macrophages as membranous CD68 positive. The proportion of Siglec-15–positive and PD-L1–positive TCs was estimated as the percentage of total PAN-CK–positive TCs, and the proportion of Siglec-15–positive macrophages was estimated as the percentage of total CD68–positive macrophages. Optimal cutoff values of high and low Siglec-15 or PD-L1 expression with respect to survival were generated using recursive partitioning analysis (RPA).^24^ The RPA method objectively divides the samples into 2 subgroups with maximum discrimination for the object variable by yielding subgroups with relatively homogeneous performance at each step. Based on RPA, we defined 5%, 50%, 0.5%, and 0.5% as the cutoff values for TC Siglec-15 positivity, macrophage Siglec-15 positivity, TC PD-L1 positivity, and macrophage PD-L1 positivity, respectively. All phenotyping and quantitative analyses were performed by an investigator blinded to the clinicopathological characteristics and clinical outcomes of the samples.

### Total RNA Isolation and Quantitative Real-Time Polymerase Chain Reaction

Total RNA was extracted from archived frozen tissues collected by the Guangdong Esophageal Cancer Institute using TRIzol reagent (Invitrogen) from 18 patients with ESCC who received esophagectomy alone, according to the manufacturer’s instructions. Aliquots (1 μg) of total RNA were reverse-transcribed into cDNA using HiScript II QRT SuperMix for quantitative polymerase chain reaction (+gDNA wiper) (Vazyme), and circulating DNAs were analyzed by quantitative real time–PCR using a 2 × RealStar SYBR Mixture (Genestar) in a CFX96 Real Time System C1000 Cycler (Bio-Rad Laboratories). Glyceraldehyde 3-phosphate dehydrogenase was used to normalize the gene expression.

### Statistical Analysis

The difference in the percentage of Siglec-15–positive TCs and Siglec-15–positive macrophages was compared using a paired *t* test. The correlation between the TC Siglec-15 positive rate and macrophage Siglec-15 or TCPD-L1 positive rate was calculated using Pearson product-moment correlation coefficient. Associations between Siglec-15 or PD-L1 expression and patient clinicopathological characteristics were examined using χ^2^ or Fisher exact test. Follow-up time was calculated from the date of surgery to the date of the last follow-up. Data analysis was conducted from January to May 2021. The Kaplan-Meier method was used to estimate the overall survival (OS) and recurrence-free survival (RFS). The log-rank test was used to examine intergroup differences, and the Cox proportional hazards regression model was used to analyze the factors associated with survival or disease recurrence (backward stepwise). Multicollinearity test was performed for variables in the multivariate regression model. Univariate and multivariate logistic regression models were used to analyze possible predictors of pCR. Variables with *P* ≤ .10 in the univariate analysis were subjected to multivariate analysis. Statistical analyses were performed using SPSS software version 24.0 (IBM Corp). *P* < .05 was considered statistically significant, and all tests were 2-tailed.

## Results

### Patient Characteristics

Patient and treatment characteristics of the 130 individuals in the primary cohort are summarized in eTable 1 in Supplement 1. The median age of this cohort was 56 years (range, 42-73 years), 108 participants (83.1%) were male, and most patients had tumors located in the middle third of the esophagus (84 [64.6%]). After neoadjuvant CRT and esophagectomy, 58 patients (44.6%) achieved a pCR based on histopathological examination.

### Siglec-15 and PD-L1 Expression in ESCC Tissues

**Figure 1.**
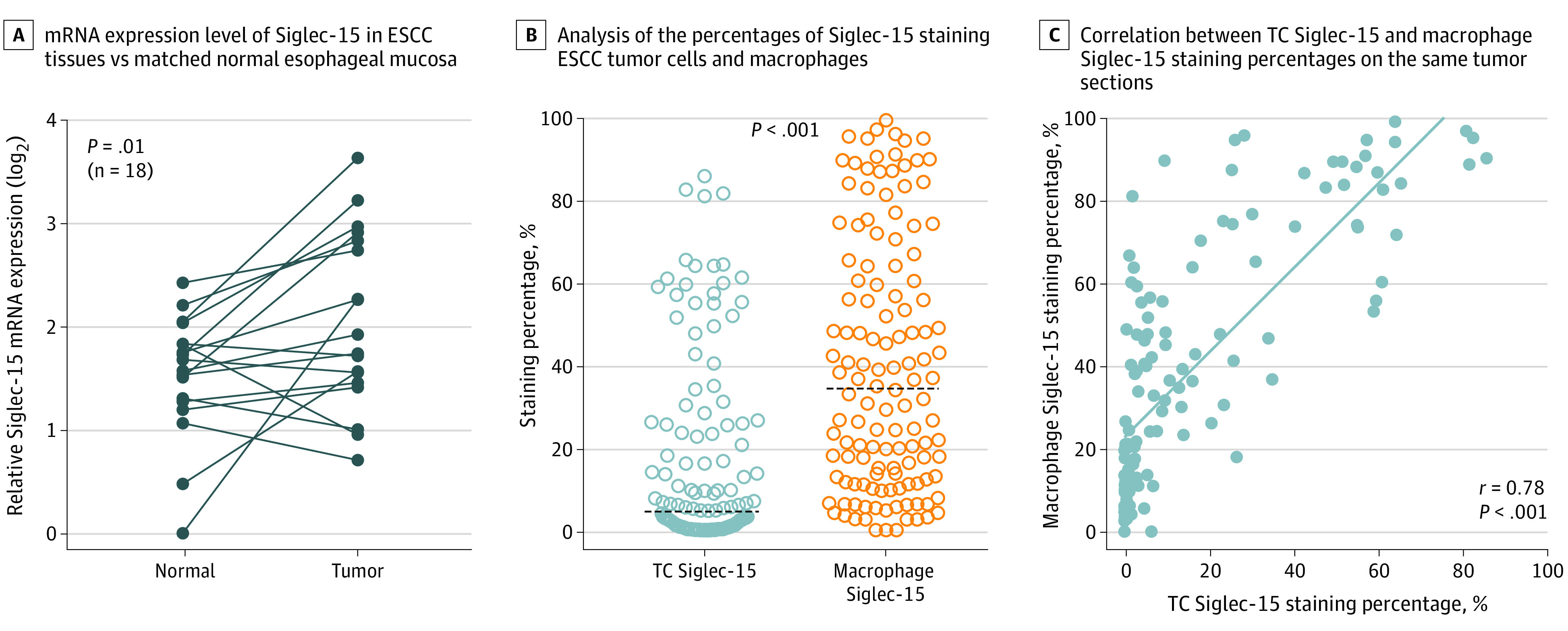
Sialic Acid–Binding Immunoglobulin-Like Lectin 15 (Siglec-15) Expression in Esophageal Squamous Cell Carcinoma (ESCC) Tissues Dots represent individual patients. B, Tumor cells (TCs) were identified as pan-cytokeratin positive, and macrophages were identified as CD68 positive. Horizontal lines indicate cutoffs.

The mRNA expression levels of Siglec-15 in 18 pairs of freshly collected ESCC tissues and matched normal esophageal mucosa were examined using RT-PCR analysis. Siglec-15 mRNA expression levels were significantly upregulated in ESCC tissues compared with those in the normal mucosa (median, 1.83 [range, 0.71–3.63] vs 1.62 [range, 0–2.42]; *P* = .01) (Figure 1A). IHC staining score analysis of surgically resected primary ESCC tumor tissue and normal esophageal mucosa indicated that Siglec-15 protein expression levels were also notably higher in tumor tissues (median: 5 [range, 0–9] vs 1 [range, 0–6]; *P* = .006) (eFigure 1A and 1B in Supplement 1).

Analysis of multiplex staining images in the primary cohort indicated that Siglec-15 and PD-L1 expressions could be detected in both TCs and tumor-associated stromal cells, including CD68^+^ macrophages (eFigure 2A in Supplement 1). Quantification of immunofluorescence signals revealed that the percentage of Siglec-15–positive macrophages was notably higher than that of Siglec-15–positive TCs (median [IQR], 34.4% [12.7%-64.3%] vs 4.8% [0.7%-25.6%]; *P* < .001) (Figure 1B). TC Siglec-15 expression was significantly and positively associated with macrophage Siglec-15 expression (*r* = 0.78; *P* < .001, Figure 1C) but not with TC PD-L1 expression (*r* = 0.19; *P* = .03) (eFigure 2B in Supplement 1).

Based on the optimal cutoff values of Siglec-15 and PD-L1 expression defined by the RPA method, 64 patients (49.2%) were defined as TC Siglec-15 positive, 43 (33.1%) as macrophage Siglec-15 positive, 70 (53.8%) as TC PD-L1 positive, and 75 (57.7%) as macrophage PD-L1 positive in the primary cohort (eFigures 2C-F in Supplement 1). The co-expression of Siglec-15 and PD-L1 on TCs and macrophages was 39 (30.0%) and 29 (22.3%), respectively, in ESCC tissues (eFigure 3A and 3B in Supplement 1). Considering that PD-L1 expression on TCs is the mainstream biomarker to guide treatment in clinical practice for patients with advanced EC, macrophage PD-L1 expression was not calculated in the following analysis.

### Different Expression Patterns of Siglec-15 and PD-L1 in ESCC Tumors

**Figure 2.**
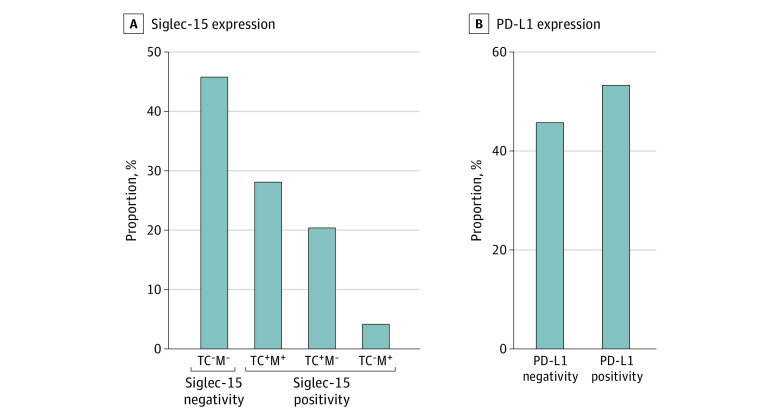
Different Expression Patterns of Sialic Acid–Binding Immunoglobulin-Like Lectin 15 (Siglec-15) and Programmed Cell Death Ligand 1 (PD-L1) in Esophageal Squamous Cell Carcinoma (ESCC) Tumors A, ESCC samples were divided into 4 groups according to their Siglec-15 expression patterns: pattern 1 (tumor cell and macrophage positive), pattern 2 (tumor cell negative and macrophage positive), pattern 3 (tumor cell positive and macrophage negative), and Pattern 4 (tumor cell and macrophage negative). B, ESCC samples were divided into 2 groups according to their PD-L1 expression patterns: PD-L1 negativity and PD-L1 positivity.

By analyzing multispectral images stained with Siglec-15, PAN-CK, and CD68, we divided ESCC samples into 4 groups according to their Siglec-15 expression patterns (eFigure 4 in Supplement 1): TC Siglec-15 positive and macrophage Siglec-15 positive (pattern 1), TC Siglec-15 negative and macrophage Siglec-15 positive (pattern 2), TC Siglec-15 positive and macrophage Siglec-15 negative (pattern 3), and TC Siglec-15 negative and macrophage Siglec-15 negative (pattern 4). In view of the significant positive correlation between TC Siglec-15 expression and macrophage Siglec-15 expression, we identified patients who had positive Siglec-15 expression on either macrophages or TCs as Siglec-15 positive. As shown in Figure 2A, 60 patients (46.2%) had tumors with negative Siglec-15 expression on both macrophages and TCs, and 70 patients (53.8%) were classified as Siglec-15 positive, including 37 (28.5%) with TC and macrophage positivity, 27 (20.8%) with TC positivity only, and 6 (4.6%) with macrophage positivity only. Meanwhile, according to PD-L1 expression on TCs (eFigure 5 in Supplement 1), patients were divided into 2 groups: 70 patients (53.8%) with PD-L1 positivity and 60 (46.2%) with PD-L1 negativity (Figure 2B).

### Association of Siglec-15 or PD-L1 Positivity With Clinicopathologic Characteristics

As listed in eTable 2 in Supplement 1, Siglec-15 positivity was significantly associated with upper tumor location. However, no significant association was observed between PD-L1 positivity and clinicopathological characteristics.

### Association of Siglec-15 and PD-L1 Positivity on pCR and Survival

Patients with Siglec-15 positivity showed a significantly higher pCR rate than those with Siglec-15 negativity (37 of 70 [52.9%] vs 21 of 60 [35.0%]; *P* = .04) (eFigure 6A in Supplement 1), and Siglec-15 positivity was the only factor significantly associated with pCR (odds ratio, 2.08; 95% CI, 1.03-4.23; *P* = .04) (eTable 3 in Supplement 1). There was no significant difference in the pCR rate between patients with PD-L1 positive and negative tumors (eFigure 6B in Supplement 1).

We further investigated the association of Siglec-15 and PD-L1 expression on survival. The median follow-up time for survivors was 63.7 months (range, 7.5-179.4 months) in the primary cohort. During the follow-up period, 46 patients (35.4%) died, and 44 (33.8%) developed locoregional or distant recurrences. The 3-year OS and RFS rates of the entire cohort were 71.9% (95% CI, 64.3%-80.5%) and 69.0% (95% CI, 60.9%-78.1%), respectively.

**Figure 3.**
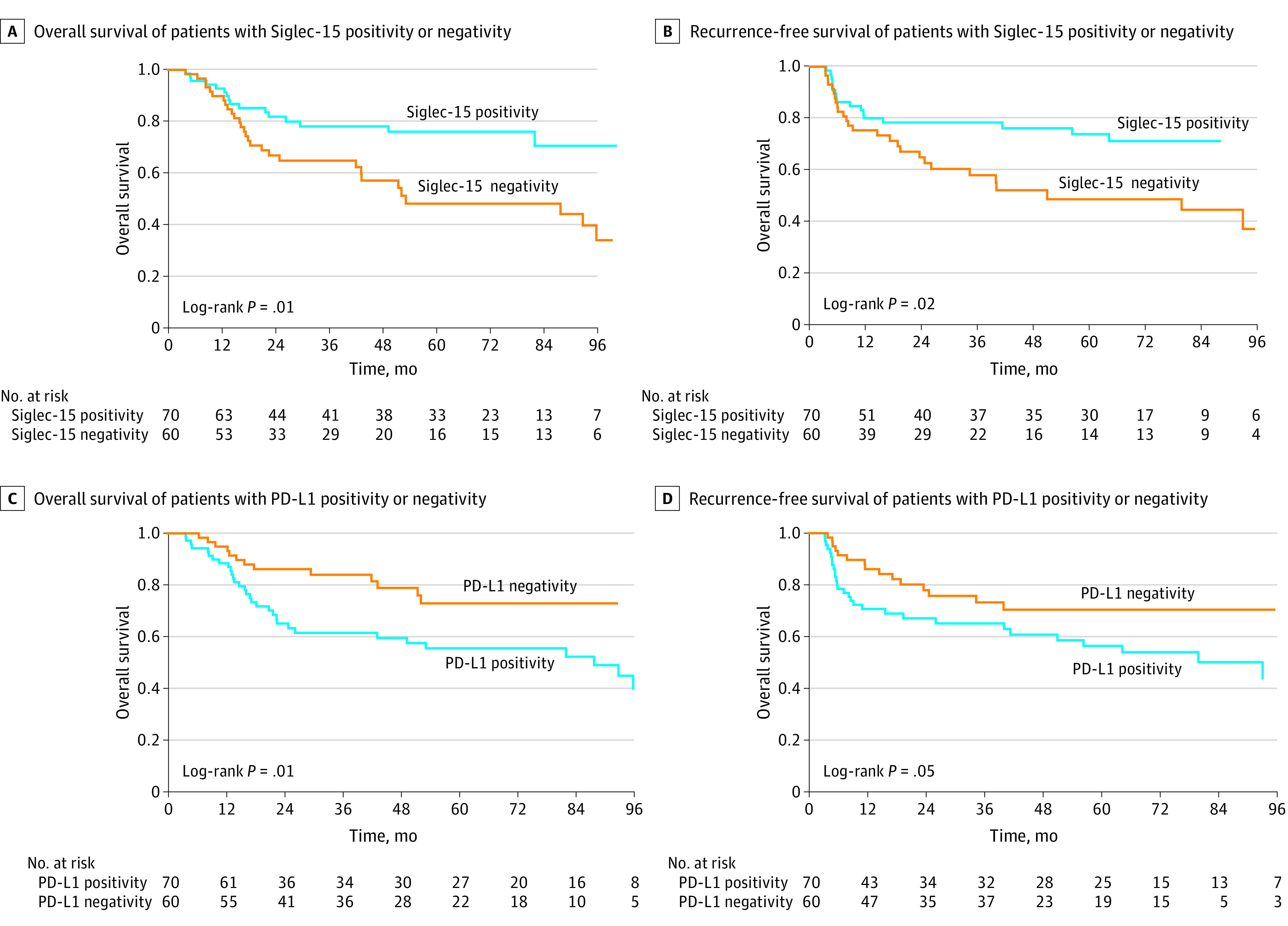
Kaplan-Meier Curves for Survival by Different Sialic Acid–Binding Immunoglobulin-Like Lectin 15 (Siglec-15) or Programmed Cell Death Ligand 1 (PD-L1) Expression Patterns Comparison of overall survival (A) and recurrence-free survival (B) between patients with Siglec-15 positivity and negativity. Comparison of overall survival (C) and recurrence-free survival (D) between patients with PD-L1 positivity or negativity.

Siglec-15 positivity was negatively associated with recurrence risk (17 [24.3%] vs 27 [45.0%]; *P* = .02), whereas patients with PD-L1 positivity showed a significantly higher recurrence rate than those with PD-L1 negativity (29 [41.4%] vs 15 [25.0%]; *P* = .048). Kaplan-Meier analysis revealed that patients with Siglec-15 positivity had better OS (hazard ratio [HR], 0.46; 95% CI, 0.25-0.85; *P* = .01) (Figure 3A) and RFS (HR, 0.48; 95% CI, 0.26-0.88; *P* = .02) (Figure 3B). In contrast, PD-L1 positivity was significantly associated with worse OS and RFS (Figure 3C and D). For variables included in the multivariate analysis, the multicollinearity test revealed no evidence of multicollinearity in the regression model, with all variance inflation factors close to 1 (eTable 4 in Supplement 1). Multivariate analysis further indicated that Siglec-15 and PD-L1 positivity were independently associated with OS and RFS, and pathologic response was independently associated with only OS (Table).

**Table.** Univariate and Multivariate Analyses for Overall Survival and Recurrence-Free Survival in the Primary Cohort

Variable	Overall survival				Recurrence-free survival			
	Univariate		Multivariate		Univariate		Multivariate	
	Patients, No./total No. (%)	*P* value	HR (95% CI)	*P* value	Patients, No./total No. (%)	*P* value	HR (95% CI)	*P* value
Age, y								
<56	29/61 (47.5)	.01	1.64 (0.88-3.04)	.12	30/61 (49.2)	.001	2.38 (1.24-4.59)	.01
≥56	17/69 (24.6)				14/69 (20.3)			
Sex								
Female	3/22 (13.6)	.02	0.32 (0.10-1.02)	.06	4/22 (13.6)	.08	0.44 (0.16-1.25)	.12
Male	43/108 (39.8)				40/108 (37.0)			
Smoking history								
Yes	35/90 (38.9)	.25	NA	NA	30/90 (33.3)	.89	NA	NA
No	11/40 (27.5)				14/40 (35.0)			
Alcohol history								
Yes	20/50 (40.0)	.45	NA	NA	19/50 (38.0)	.47	NA	NA
No	26/80 (32.5)				25/80 (31.3)			
Performance status								
0	24/71 (33.8)	.67	NA	NA	22/71 (31.0)	.86	NA	NA
1-2	22/59 (37.3)				22/59 (37.3)			
Weight loss								
<10%	43/118 (36.4)	.55	NA	NA	42/118 (35.6)	.31	NA	NA
≥10%	3/12 (25.0)				2/12 (16.7)			
Histologic grade								
1 or 2	37/97 (38.1)	.46	NA	NA	35/97 (36.1)	.52	NA	NA
3	9/33 (27.3)				9/33 (27.3)			
Tumor location								
Upper or middle	35/97 (36.1)	.78	NA	NA	33/97 (34.0)	.94	NA	NA
Distal	11/33 (33.3)				11/33 (33.3)			
Primary tumor length, cm								
≤5	17/63 (27.0)	.05	0.70 (0.38-1.28)	.25	19/63 (30.2)	.26	NA	NA
>5	29/67 (43.3)				25/67 (37.3)			
Clinical T stage								
T1-2	8/24 (33.3)	.88	NA	NA	8/24 (33.3)	.73	NA	NA
T3-4	38/106 (35.8)				36/106 (34.0)			
Clinical N stage								
N0	4/10 (40.0)	.87	NA	NA	4/10 (40.0)	.92	NA	NA
N1-3	42/120 (35.0)				40/120 (33.3)			
Chemotherapy regimen								
Cisplatin/vinorelbine	31/84 (36.9)	.43	NA	NA	29/84 (34.5)	.29	NA	NA
Cisplatin/fluorouracil or cisplatin/taxane	15/46 (32.6)				15/46 (32.6)			
Radiation dose, Gy								
≤40	41/97 (42.3)	.28	NA	NA	35/97 (36.1)	.67	NA	NA
>40	5/33 (15.2)				9/33 (27.3)			
Radiotherapy modality								
3DCRT	41/96 (42.7)	.22	NA	NA	36/96 (37.5)	.79	NA	NA
IMRT	5/34 (14.7)				8/34 (23.5)			
Treatment era								
2002-2012	35/68 (51.5)	.17	NA	NA	29/68 (42.6)	.86	NA	NA
2013-2017	11/62 (17.7)				15/62 (24.2)			
Pathologic response								
pCR	14/58 (24.1)	.02	0.52 (0.27-0.98)	.04	14/58 (24.1)	.02	0.53 (0.27-1.03)	.06
No pCR	32/72 (44.4)				30/72 (41.7)			
Siglec-15								
Positive	17/70 (24.3)	.01	0.44 (0.24-0.82)	.009	17/70 (24.3)	.02	0.51 (0.27-0.95)	.04
Negative	29/60 (48.3)				27/60 (45.0)			
PD-L1								
Positive	33/70 (47.1)	.01	2.81 (1.46-5.41)	.002	29/70 (41.4)	.048	1.97 (1.03-3.78)	.04
Negative	13/60 (21.7)				15/60 (25.0)			

Abbreviations: 3DCRT, 3-dimensional conformal radiation therapy; HR, hazard ratio; IMRT, intensity-modulated radiation therapy; NA, not applicable; pCR, pathologic complete response; Siglec-15, sialic acid–binding immunoglobulin-like lectin 15; PD-L1, programmed cell death ligand 1.

### Stratification Analysis

**Figure 4.**
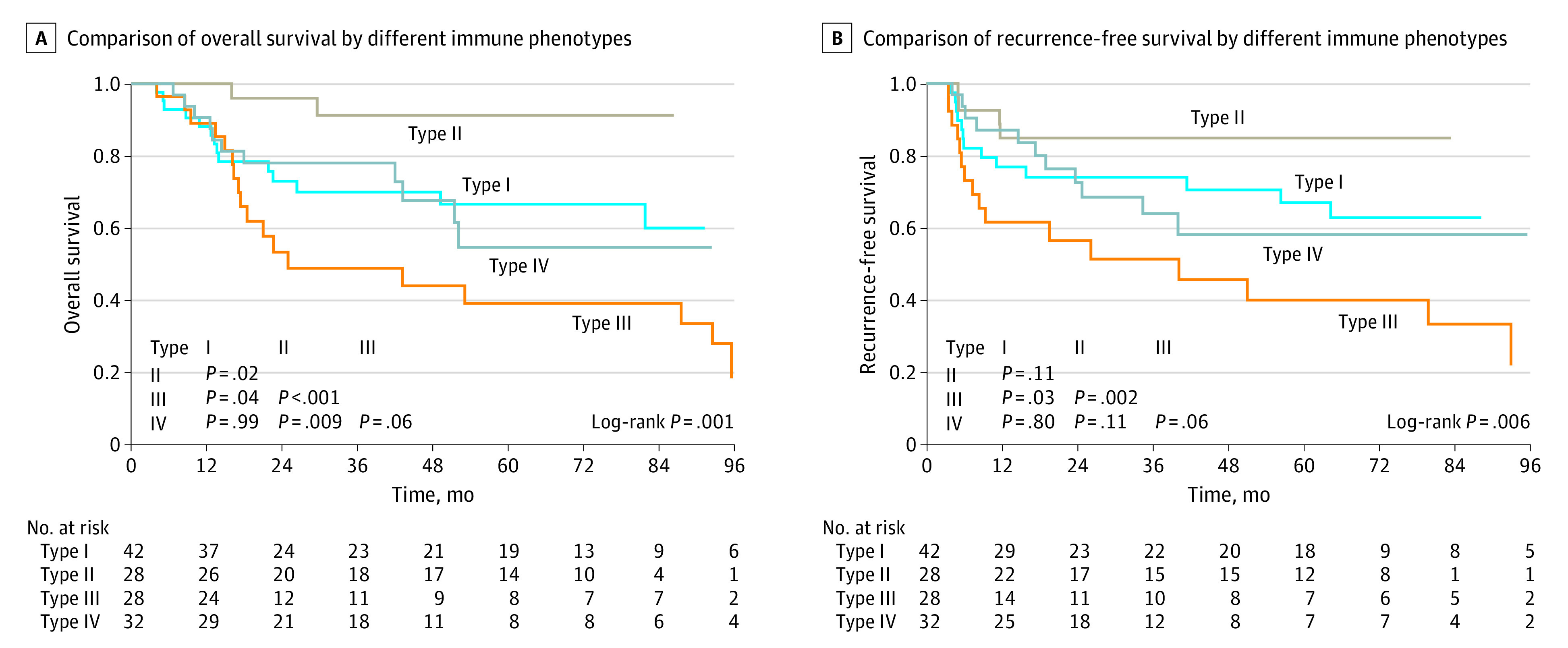
Association of Different Immune Phenotypes of Sialic Acid–Binding Immunoglobulin-Like Lectin 15 (Siglec-15) and Programmed Cell Death Ligand 1 (PD-L1) With Survival Type I was defined as Siglec-15 positivity with PD-L1 positivity (42 of 130 patients [32.4%]); type II, Siglec-15 positivity with PD-L1 negativity (28 patients [21.5%]); type III, Siglec-15 negativity with PD-L1 positivity (28 patients [21.5%]); and type IV, Siglec-15 negativity with PD-L1 negativity (32 patients [24.6%]).

Considering the opposite significance of Siglec-15 positivity and PD-L1 positivity, we classified the 130 patients with ESCC in the primary cohort into 4 immune phenotypes (eFigure 7 in Supplement 1): type I (Siglec-15 positivity with PD-L1 positivity; 42 patients [32.4%]), type II (Siglec-15 positivity with PD-L1 negativity; 28 patients [21.5%]), type III (Siglec-15 negativity with PD-L1 positivity; 28 patients [21.5%]), and type IV (Siglec-15 negativity with PD-L1 negativity; 32 patients [24.6%]), with corresponding recurrence rates of 31.0% (13 patients), 14.3% (4 patients), 57.1% (16 patients), and 34.4% (11 patients), respectively (*P* = .008). Survival analysis showed that patients with type III had the worst outcomes, whereas patients with type II had the most favorable OS (Figure 4A) and RFS (Figure 4B). The 5-year RFS rates for types I, II, III, and IV were 66.9% (95% CI, 53.0%-84.6%), 84.9% (95% CI, 72.3%-99.7%), 39.9% (95% CI, 23.5%-67.6%), and 58.1% (95% CI, 41.3%-81.8%), respectively (*P* = .006).

### Independent Validation

Patient characteristics of the independent validation cohort are listed in eTable 5 in Supplement 1. Of these 55 patients, 12 (21.8%) experienced recurrence after surgery at a median follow-up time of 15.2 months (range, 0.4-25.9 months).

According to the cutoff values defined in the primary cohort, 29 patients (52.7%) patients were defined as TC Siglec-15 positive, 21 (38.2%) as macrophage Siglec-15 positive, and 27 (49.1%) as TC PD-L1 positive (eFigure 8A-8C in Supplement 1). Patients with Siglec-15 positivity had a higher pCR rate than those with Siglec-15 negativity, but the difference was not statistically significant (50.0% vs 26.1%; *P* = .07) (eFigure 9A in Supplement 1). Kaplan-Meier curves showed that patients with Siglec-15 positivity had a better RFS, and patients with PD-L1 positivity had a worse RFS (eFigure 9B and 9C in Supplement 1). Further stratification analysis confirmed the survival difference among the 4 immune phenotypes, with 1-year RFS rates of 81.5% (95% CI, 61.1%-100%), 87.5% (95% CI, 67.3%-100%), 30.0% (95% CI, 11.6%-77.3%), and 83.1% (95% CI, 64.1%-100%) for types I, II, III, and IV, respectively (*P* = .009) (eFigure 10 in Supplement 1).

## Discussion

Immunotherapy has been proposed as the fourth method of cancer treatment after surgery, chemotherapy, and radiotherapy.^25,26^ Blockade of the PD-1/PD-L1 pathway using specific monoclonal antibodies is widely accepted as a successful strategy for the normalization of cancer immunotherapy.^27,28,29,30,31,32^ However, treatments targeting the PD-1/PD-L1 pathway cannot be applied to all patients. Siglec-15 has recently been identified as an immunotherapy target. In this study, we found that Siglec-15 was detectable in both TCs and tumor-associated macrophages in patients with ESCC. More importantly, the expression of Siglec-15 was associated with pCR and could be considered an independent prognostic factor for patients with ESCC after neoadjuvant CRT.

Siglec-15 has been identified as a pivotal regulator of bone remodeling and osteoclast differentiation, and Siglec-15–targeting therapy may have potential implications in the treatment of osteoporosis.^33,34,35^ Its role in dampening antitumor immunity was recently revealed by Wang et al^18^ using the genome-scale T-Cell Activity Array. Analysis of the GTEx and TCGA databases indicated that the mRNA expression of Siglec-15 is significantly upregulated in several malignant neoplasms, such as lung adenocarcinoma, head and neck squamous cell carcinoma, and EC, compared with their respective normal tissues.^19^ Immunohistochemical staining of Siglec-15 in primary tumor tissues from patients with non–small cell lung cancer (NSCLC) and diffuse large B-cell lymphoma confirmed Siglec-15 protein expression in both TCs and macrophages.^18,20^ Interestingly, the expression of Siglec-15 in macrophages is inhibited by interferon-γ 18, a dominant cytokine required for PD-L1 induction, suggesting different expression patterns of Siglec-15 and PD-L1 in tumors. In the present study, we found that Siglec-15 was more frequently expressed on macrophages than on TCs, and the co-expression of Siglec-15 and PD-L1 on TCs and macrophages was 30.0% and 22.3%, respectively, in ESCC tissues. Our results are consistent with those of the study reported by Fudaba et al,^20^ in which the co-expression of Siglec-15 and PD-L1 was detected in approximately 20% of macrophages in lymphoma. However, Wang et al^18^ and Maria et al^36^ reported that the expression of Siglec-15 is mutually exclusive of PD-L1 in NSCLC, with only approximately 3% of specimens positive for both markers. These inconsistent results suggest that the expression patterns of immune markers vary significantly across different cancer types.

Growing evidence highlights the prognostic value of Siglec-15 in human cancers. Bioinformatics analysis using next-generation sequencing data from public databases has shown that Siglec-15 has variable prognostic significance in diverse types of cancer.^19^ High expression of Siglec-15 indicated better OS for NSCLC, thyroid carcinoma, and bladder urothelial carcinoma, whereas the opposite results were observed in renal clear cell carcinoma, sarcoma, and pancreatic adenocarcinoma. Siglec-15 expression even had contrasting association in different subtypes of breast cancer (*BRCA*); it was associated with longer OS and RFS for *BRCA* luminal A and *BRCA* luminal B but worse OS for *BRCA* basal.^19^ In our study, Siglec-15 positivity on either TCs or macrophages was associated with favorable outcomes in patients with ESCC. The contrasting significance of Siglec-15 expression in different cancer types may be because of intertumoral heterogeneity and different immune infiltration properties. For example, Siglec-15 was positively associated with CD8+ T cells in adrenocortical carcinoma but negatively associated with CD8+ T cells in *BRCA* basal breast cancer.^19^ The correlation between the composition of immune cells and Siglec-15 in ESCC remains unclear, which warrants further investigation. Our study also indicated for the first time that we are aware of that Siglec-15 positivity was significantly associated with pCR after neoadjuvant CRT, suggesting that Siglec-15 may participate in the regulation of radiosensitivity in ESCC. Previous studies reported that Siglec-15 could affect several cancer-related pathways, including PI3K-Akt, MAPK, Hippo, and apoptosis.^19,37^ Whether Siglec-15 could regulate radiosensitivity and its exact mechanisms remain to be elucidated, which presents new challenges for Siglec-15 studies.

The prognostic role of PD-L1 on TCs in patients with EC treated with esophagectomy alone remains controversial. Yagi et al^38^ found that PD-L1 positivity was significantly associated with worse OS, while the opposite result was reported by Hatogai et al.^39^ Our previous report found that PD-L1 positivity in TCs of pre-CRT ESCC tissues was significantly associated with a lower pCR rate and worse RFS.^40^ However, the current study failed to find a significant association between PD-L1 expression and pCR. The discrepancies between studies might be partially explained by the differences in the experimental methods and cutoff values.

We further explored different immune phenotypes based on the combined expression patterns of Siglec-15 and PD-L1. Patients with type III ESCC (ie, Siglec-15 negativity and PD-L1 positivity) had the worst outcomes and were most likely to benefit from a single anti–PD-1/PD-L1 blocker. The treatment strategy of combining anti–PD-1 and anti–Siglec-15 blockers might be appropriate for patients with type I disease (Siglec-15 positivity and PD-L1 positivity). For patients with type IV (Siglec-15 negativity and PD-L1 negativity), additional immune checkpoint molecules need to be detected to guide treatment. To date, trials of Siglec-15–targeted immunotherapy are rare. Preliminary findings of anti–Siglec-15 (NC318) monotherapy exhibited encouraging antitumor efficacy in a phase 1 study.^36^ Future studies are needed to identify the factors associated with anti–Siglec-15 treatment response and explore the best combination strategy, including chemotherapy, radiotherapy, and different immune cell modulation reagents.

### Limitations

This study has several limitations. First, the results may be affected by a selection bias owing to the retrospective nature of the study conducted in a single institution. Second, the clinical significance of macrophage PD-L1 expression was not analyzed in this study, which may have introduced bias to the results. Third, we used binary classification of Siglec-15 and PD-L1 expression, which may lead to the loss of some information. In addition, although the major findings were reproducible in the validation cohort, independent external validation is required in the future.

## Conclusions

In this study, the expression of Siglec-15 was associated with better pathological response and favorable survival outcomes in patients with ESCC after neoadjuvant CRT, suggesting that Siglec-15 could be used as a novel biomarker to identify candidates who may benefit from immunotherapy combined with CRT. Further prospective studies are warranted to confirm the role of Siglec-15 as a biomarker for pCR as well as prognostic phenotypes.

## Notes

Supplement 1.
eMethods.
**eFigure 1. **IHC Staining of Siglec-15 in Normal Esophageal Mucosa and Primary ESCC Tissues **eFigure 2. **Staining Percentage of Siglec-15 and PD-L1 in ESCC Tissues **eFigure 3. **The Co-Expression of Siglec-15 and PD-L1 in the Primary ESCC Cohort **eFigure 4. **Different Expression Patterns of Siglec-15 in ESCC Tissues **eFigure 5.** Different Expression Patterns of PD-L1 in ESCC Tissues **eFigure 6.** Comparison of Pathologic Complete Response Rates by Different Siglec-15 or PD-L1 Expression Patterns **eFigure 7.** Different Immune Phenotypes of Siglec-15 and PD-L1 in ESCC Tissues **eFigure 8. **Staining Percentage of Siglec-15 and PD-L1 in the Independent Validation Cohort **eFigure 9. **Comparison of Pathologic Complete Response Rates and Recurrence-Free Survival by Different Siglec-15 or PD-L1 Expression Patterns in the Independent Validation Cohort **eFigure 10. **Stratification Analysis of the Survival Difference Among the 4 Immune Phenotypes in the Independent Validation Cohort **eTable 1.** Patient Characteristics in the Primary Cohort **eTable 2.** Association of Siglec-15 and PD-L1 Expression With Patient Clinicopathological Characteristics in the Primary Cohort **eTable 3. **Univariate and Multivariate Analyses for Variables Associated With Pathologic Complete Response in the Primary Cohort **eTable 4. **Multicollinearity Test for Variables in the Multivariate Regression Model **eTable 5.** Patient Characteristics in the Independent Validation Cohort

Supplement 2.
Data Sharing Statement
