# Supplementary material for: Association of Sialic Acid–Binding Immunoglobulin-Like Lectin 15 With Phenotypes in Esophageal Squamous Cell Carcinoma in the Setting of Neoadjuvant Chemoradiotherapy
Source: JAMA Netw Open. 2023 Jan 17;6(1):e2250965. doi: 10.1001/jamanetworkopen.2022.50965 (PMC9856737; doi:10.1001/jamanetworkopen.2022.50965)
Supplement: Supplement 1. — eMethods. eFigure 1. IHC Staining of Siglec-15 in Normal Esophageal Mucosa and Primary ESCC Tissues eFigure 2. Staining Percentage of Siglec-15 and PD-L1 in ESCC Tissues eFigure 3. The Co-Expression of Siglec-15 and PD-L1 in the Primary ESCC Cohort eFigure 4. Different Expression Patterns of Siglec-15 in ESCC Tissues eFigure 5. Different Expression Patterns of PD-L1 in ESCC Tissues eFigure 6. Comparison of Pathologic Complete Response Rates by Different Siglec-15 or PD-L1 Expression Patterns eFigure 7. Different Immune Phenotypes of Siglec-15 and PD-L1 in ESCC Tissues eFigure 8. Staining Percentage of Siglec-15 and PD-L1 in the Independent Validation Cohort eFigure 9. Comparison of Pathologic Complete Response Rates and Recurrence-Free Survival by Different Siglec-15 or PD-L1 Expression Patterns in the Independent Validation Cohort eFigure 10. Stratification Analysis of the Survival Difference Among the 4 Immune Phenotypes in the Independent Validation Cohort eTable 1. Patient Characteristics in the Primary Cohort eTable 2. Association of Siglec-15 and PD-L1 Expression With Patient Clinicopathological Characteristics in the Primary Cohort eTable 3. Univariate and Multivariate Analyses for Variables Associated With Pathologic Complete Response in the Primary Cohort eTable 4. Multicollinearity Test for Variables in the Multivariate Regression Model eTable 5. Patient Characteristics in the Independent Validation Cohort [file jamanetwopen-e2250965-s001.pdf]

Supplemental Online Content

Zhou S, Wang Y, Zhang R, et al. Association of sialic acid–binding immunoglobulin-like lectin 15 with phenotypes in esophageal squamous cell carcinoma in the setting of neoadjuvant chemoradiotherapy. *JAMA Netw Open.* 2023;6(1):e2250965. doi:10.1001/jamanetworkopen.2022.50965

eMethods.

eFigure 1. IHC Staining of Siglec-15 in Normal Esophageal Mucosa and Primary ESCC Tissues

eFigure 2. Staining Percentage of Siglec-15 and PD-L1 in ESCC Tissues

eFigure 3. The Co-Expression of Siglec-15 and PD-L1 in the Primary ESCC Cohort

eFigure 4. Different Expression Patterns of Siglec-15 in ESCC Tissues

eFigure 5. Different Expression Patterns of PD-L1 in ESCC Tissues

eFigure 6. Comparison of Pathologic Complete Response Rates by Different Siglec-15 or PD-L1 Expression Patterns

eFigure 7. Different Immune Phenotypes of Siglec-15 and PD-L1 in ESCC Tissues

eFigure 8. Staining Percentage of Siglec-15 and PD-L1 in the Independent Validation Cohort

eFigure 9. Comparison of Pathologic Complete Response Rates and Recurrence-Free Survival by Different Siglec-15 or PD-L1 Expression Patterns in the Independent Validation Cohort

eFigure 10. Stratification Analysis of the Survival Difference Among the 4 Immune Phenotypes in the Independent Validation Cohort

eTable 1. Patient Characteristics in the Primary Cohort

eTable 2. Association of Siglec-15 and PD-L1 Expression With Patient Clinicopathological Characteristics in the Primary Cohort

eTable 3. Univariate and Multivariate Analyses for Variables Associated With Pathologic Complete Response in the Primary Cohort

eTable 4. Multicollinearity Test for Variables in the Multivariate Regression Model

eTable 5. Patient Characteristics in the Independent Validation Cohort

This supplemental material has been provided by the authors to give readers additional information about their work.

© 2023 Zhou S et al. *JAMA Network Open.*

eMethods

Antibodies

The primary antibodies used were as follows: rabbit anti-human Siglec-15 polyclonal antibody (PA5-72765, Thermo Fisher Scientific), rabbit anti-human PD-L1 monoclonal antibody (ab228415, Abcam), mouse anti-human CD68 monoclonal antibody (ab955, Abcam), and mouse anti-human pan-cytokeratin monoclonal antibody (ab215838, Abcam).

Multiplex staining and multispectral imaging

Each FFPE slide was sequentially stained using four different primary antibodies, followed by incubation with horseradish peroxidase (HRP)-conjugated secondary antibodies and tyramide signal amplification (TSA) reagents. After each round of TSA incubation, microwave heat-induced antigen retrieval was performed using a citric acid solution (pH 6.0). The 4′-6′-diamidino-2-phenylindole (DAPI, Sigma-Aldrich) was used to highlight nuclei after four sequential rounds of staining. In the staining process, covalent binding between the marker protein and fluorophore using TSA was mediated by an HRP-conjugated secondary antibody.

Multiplex-stained slides were imaged using the Mantra System (PerkinElmer), which establishes an image cube by capturing the fluorescent spectra at 20-nm wavelength intervals from 420 to 720 nm. Ten fields of view in the tumor area of each section were randomly acquired at 200× multispectral images to generate a group of raw multispectral images for further quantitative digital analysis.

© 2023 Zhou S et al. *JAMA Network Open.*

eFigure 1. IHC Staining of Siglec-15 in Normal Esophageal Mucosa and Primary ESCC Tissues

(A) Representative images of IHC staining of Siglec-15 in surgically resected normal esophageal mucosa and primary ESCC tissue. (B) Quantification of Siglec-15 staining score in normal and ESCC samples.

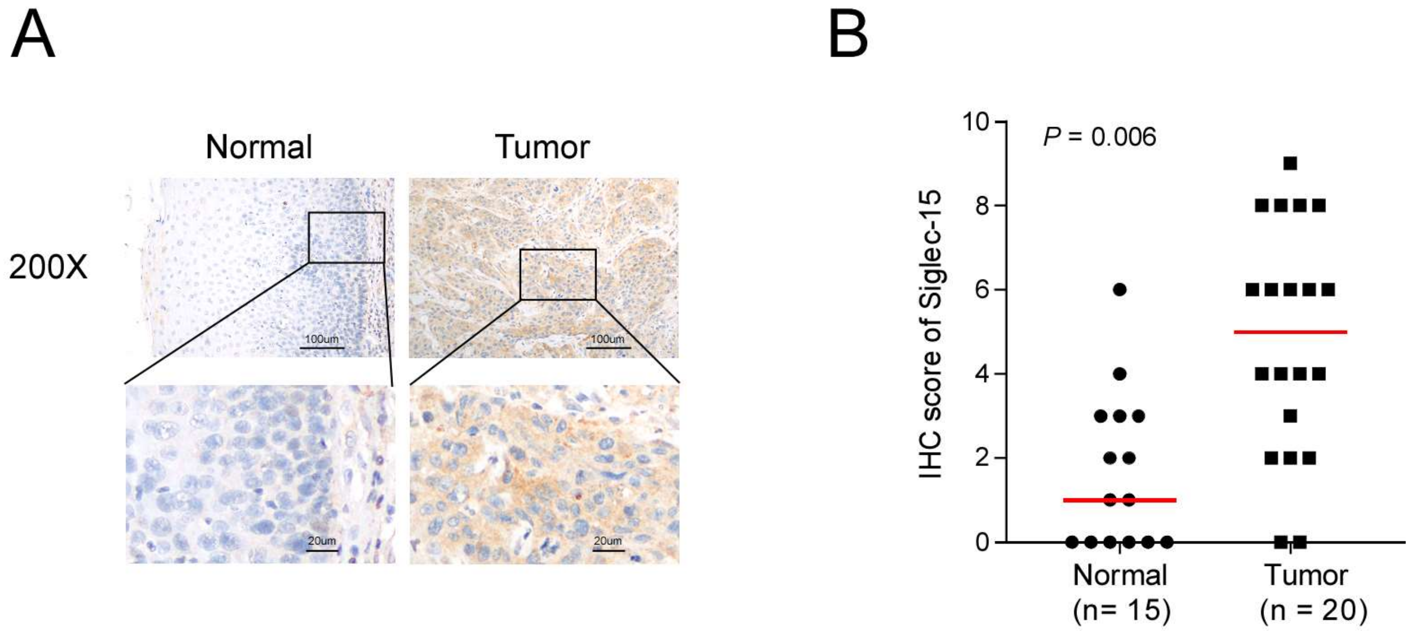

© 2023 Zhou S et al. *JAMA Network Open.*

eFigure 2. Staining Percentage of Siglec-15 and PD-L1 in ESCC Tissues

(A) Representative images of ESCC tissue section with multiplex immunofluorescence staining with the indicated markers. Scale bar, 50μm (B) The correlation between Tc-Siglec-15 staining percentage and Tc-PD-L1 staining percentage on the same tumor sections. Pearson r score and *P* values are shown. (C-F) Distribution of Tc-Siglec-15 (C), Mφ-Siglec-15 (D), Tc-PD-L1 (E) and Mφ-PD-L1 (F) staining percentage in the primary ESCC cohort. Numbers and percentage of patient cases with positive Siglec-15 staining are shown.

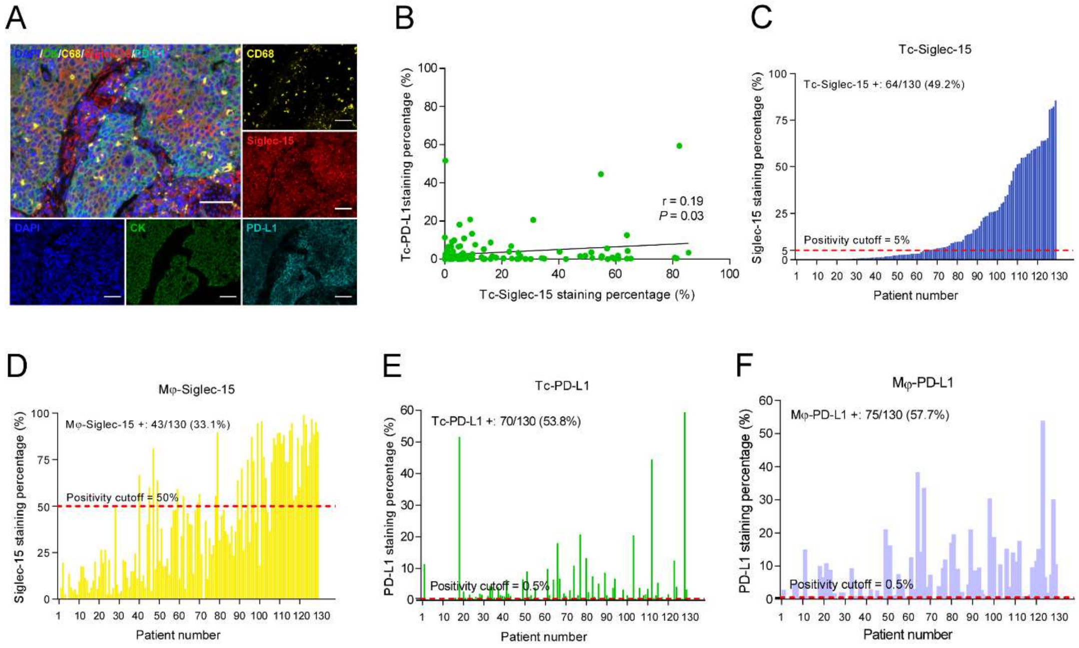

© 2023 Zhou S et al. *JAMA Network Open.*

eFigure 3. The Co-Expression of Siglec-15 and PD-L1 in the Primary ESCC Cohort

The co-expression of Siglec-15 and PD-L1 on tumor cells (A) and macrophages (B) in the primary ESCC cohort.

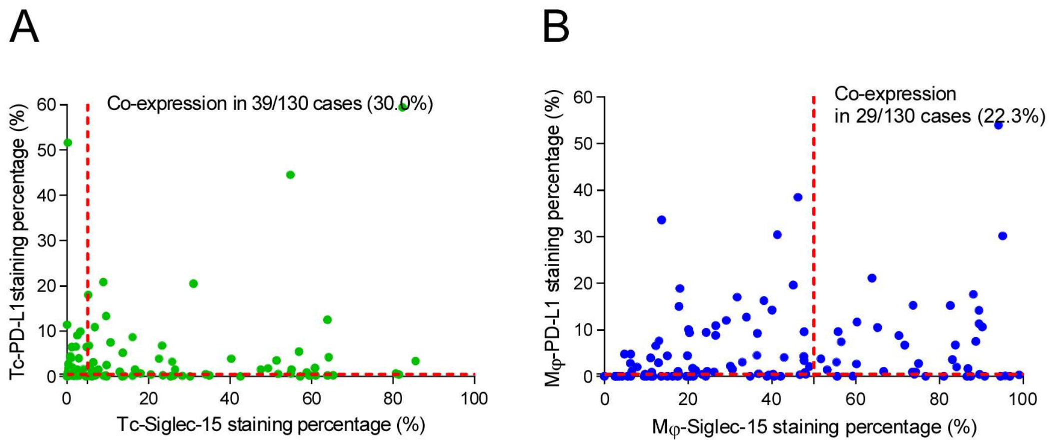

© 2023 Zhou S et al. *JAMA Network Open.*

eFigure 4. Different Expression Patterns of Siglec-15 in ESCC Tissues

Representative images of ESCC tissue sections with Siglec-15 expression Pattern 1 ($Tc^+M\varphi^+$), Pattern 2 ($Tc^-M\varphi^+$), Pattern 3 ($Tc^+M\varphi^-$), and Pattern 4 ($Tc^-M\varphi^-$). Scale bar, 50μm.

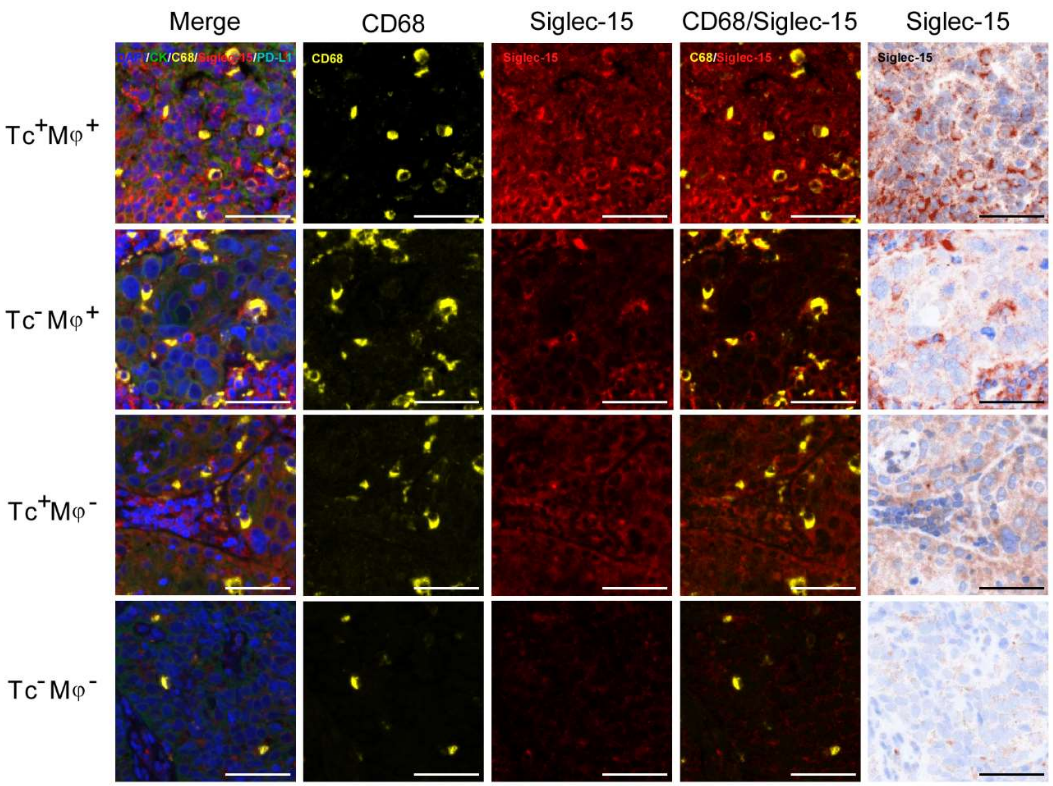

© 2023 Zhou S et al. *JAMA Network Open.*

eFigure 5. Different Expression Patterns of PD-L1 in ESCC Tissues

Representative images of ESCC tissue sections with PD-L1 positivity and negativity. Scale bar, 50µm.

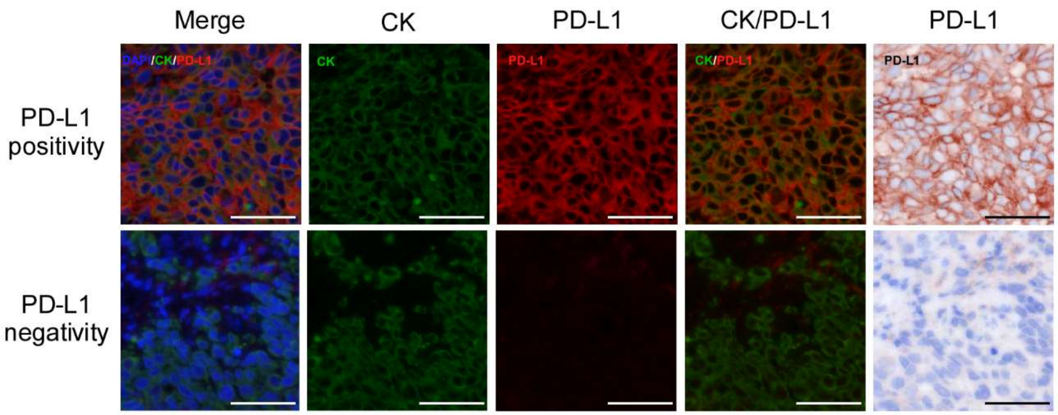

© 2023 Zhou S et al. *JAMA Network Open.*

eFigure 6. Comparison of Pathologic Complete Response Rates by Different Siglec-15 or PD-L1 Expression Patterns

(A) Comparison of pathologic complete response rates between patients with Siglec-15 positivity or negativity. (B) Comparison of pathologic complete response rates between patients with PD-L1 positivity or negativity.

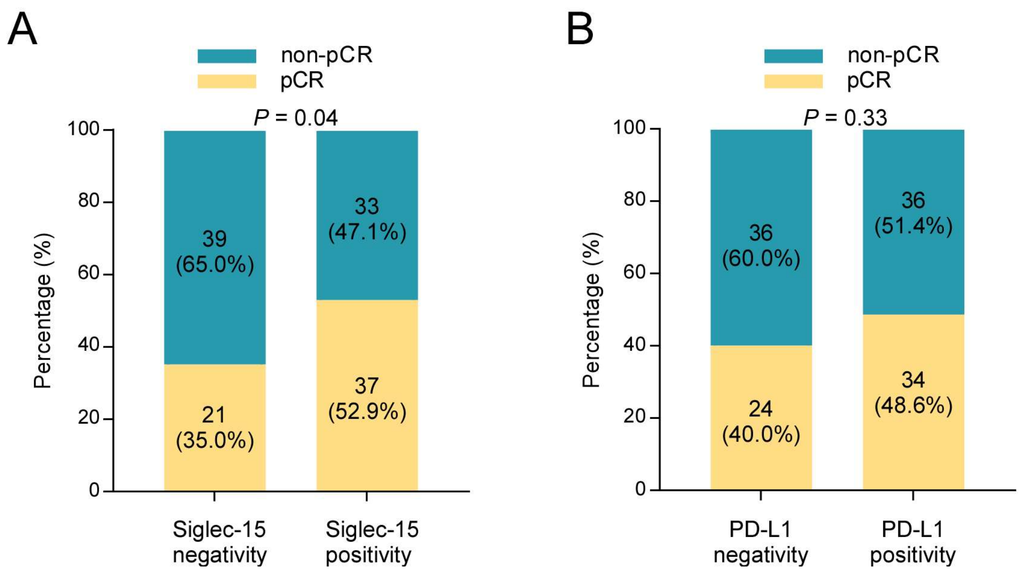

© 2023 Zhou S et al. *JAMA Network Open.*

eFigure 7. Different Immune Phenotypes of Siglec-15 and PD-L1 in ESCC Tissues

Representative images of ESCC tissue sections with immune Type I (Siglec-15 positivity/PD-L1 positivity), Type II (Siglec-15 positivity/PD-L1 negativity), Type III (Siglec-15 negtivity/PD-L1 positivity), and Type IV (Siglec-15 negativity /PD-L1 negativity). Scale bar, 50μm.

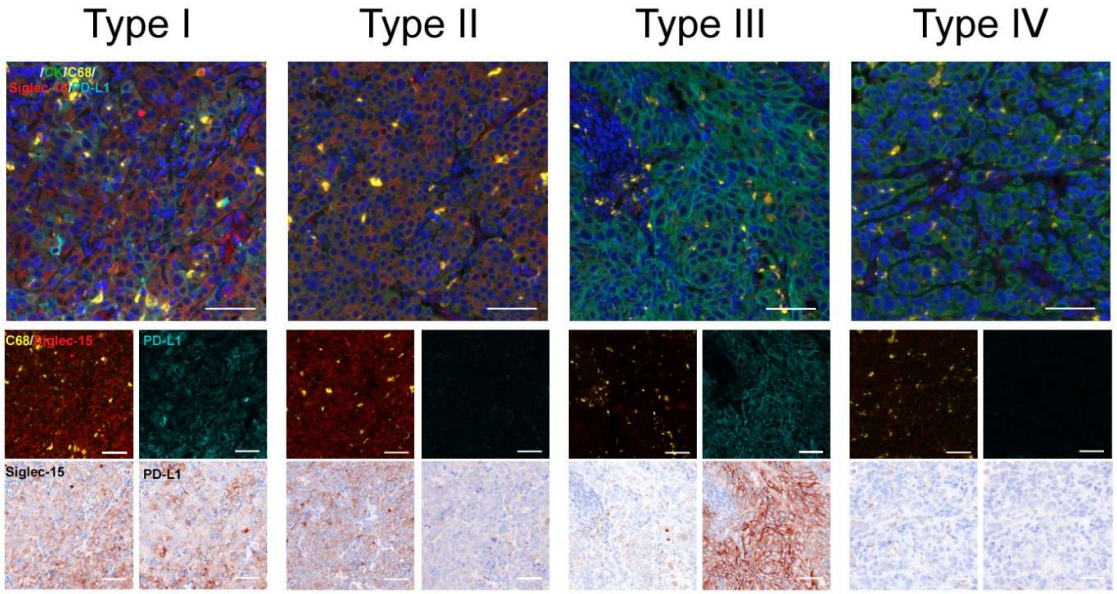

© 2023 Zhou S et al. *JAMA Network Open.*

eFigure 8. Staining Percentage of Siglec-15 and PD-L1 in the Independent Validation Cohort

Distribution of Tc-Siglec-15 (A), Mφ-Siglec-15 (B) and Tc-PD-L1 (C) staining percentage in the independent validation cohort.

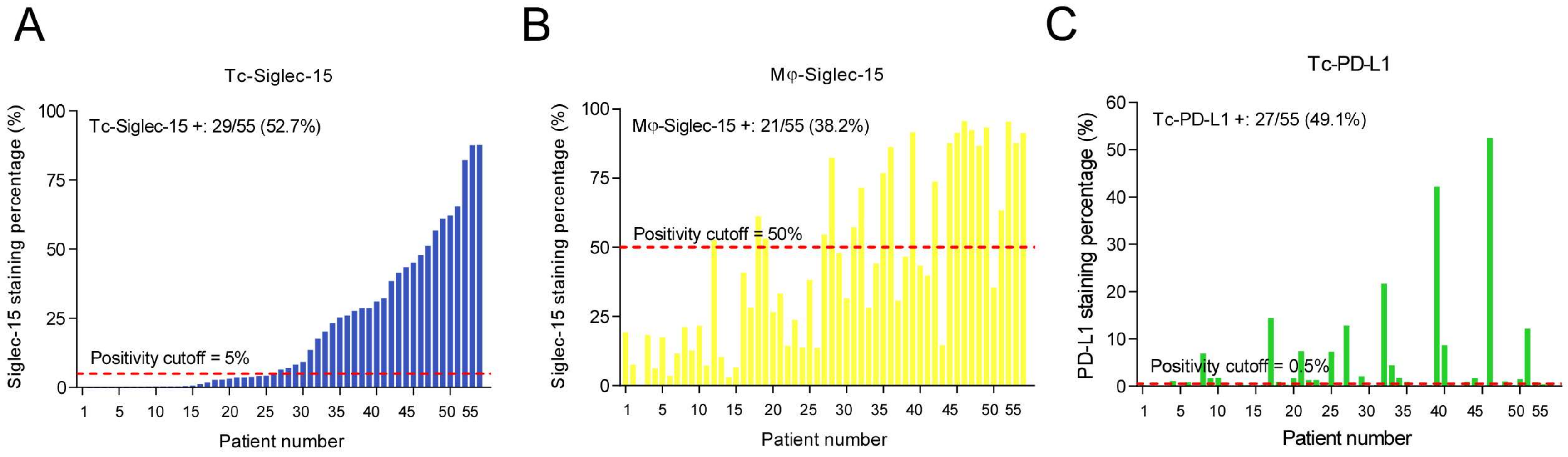

© 2023 Zhou S et al. *JAMA Network Open.*

eFigure 9. Comparison of Pathologic Complete Response Rates and Recurrence-Free Survival by Different Siglec-15 or PD-L1 Expression Patterns in the Independent Validation Cohort

(A) Comparison of pathologic complete response rates between patients with Siglec-15 positivity or negativity in the independent validation cohort. (B) Comparison of recurrence-free survival between patients with Siglec-15 positivity or negativity in the independent validation cohort. (C) Comparison of recurrence-free survival between patients with PD-L1 positivity or negativity in the independent validation cohort.

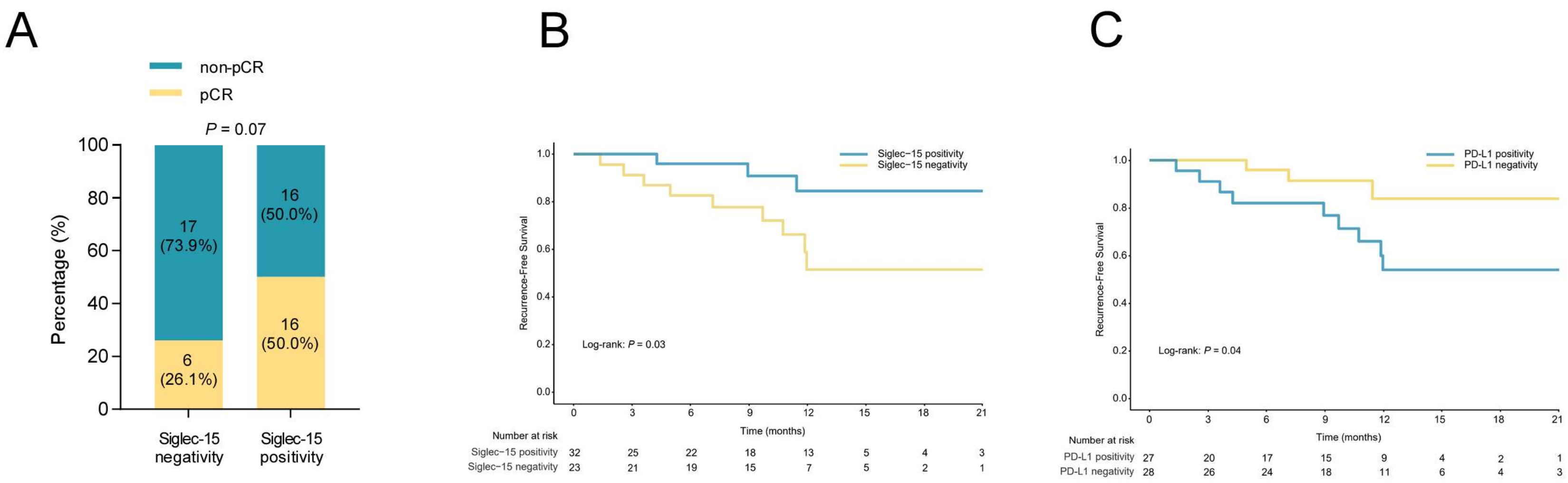

© 2023 Zhou S et al. *JAMA Network Open.*

eFigure 10. Stratification Analysis of the Survival Difference Among the 4 Immune Phenotypes in the Independent Validation Cohort

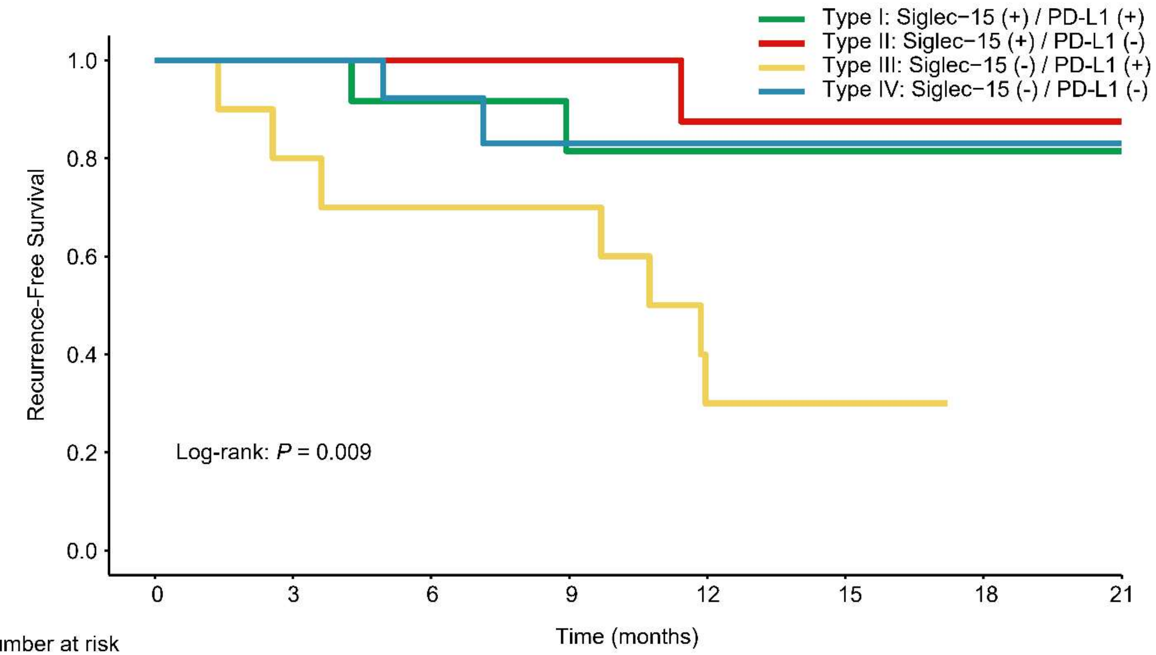

Number at risk

Type I	17	12	10	8	6	2	2	1
Type II	15	13	12	10	7	3	2	2
Type III	10	8	7	7	3	2	0	0
Type IV	13	13	12	8	4	3	2	1

© 2023 Zhou S et al. *JAMA Network Open.*

eTable 1. Patient Characteristics in the Primary Cohort

Characteristic	Total (n = 130), %
Age (years)	
<56	61 (46.9)
≥56	69 (53.1)
Sex	
Male	108 (83.1)
Female	22 (16.9)
Smoking history	
Yes	90 (69.2)
No	40 (30.8)
Alcohol history	
Yes	50 (38.5)
No	80 (61.5)
Performance status	
0	71 (54.6)
1–2	59 (45.4)
Weight loss	
<10%	118 (90.8)
≥10%	12 (9.2)
Histologic grade	
Gx/1/2	97 (74.6)
G3	33 (25.4)
Tumor location	
Upper	13 (10.0)
Middle	84 (64.6)

© 2023 Zhou S et al. *JAMA Network Open.*

Distal	33 (25.4)
Primary tumor length	
≤5 cm	63 (48.5)
>5 cm	67 (51.5)
Clinical T stage	
T1-2	24 (18.5)
T3-4	106 (81.5)
Clinical N stage	
N0	10 (7.7)
N1-3	120 (92.3)
Clinical TNM stage	
II	28 (21.5)
III	92 (70.8)
IVa	10 (7.7)
Chemotherapy regimen	
Cisplatin/vinorelbine	84 (64.6)
Cisplatin/fluorouracil	11 (8.5)
Cisplatin/taxane	35 (26.9)
Radiation dose (Gy)	
Median (range)	40.0 (40.0–45.5)
Radiotherapy modality	
3DCRT	96 (73.8)
IMRT	34 (26.2)
Surgical procedure	
Ivor-Lewis esophagectomy	130 (100.0)
Treatment era	

© 2023 Zhou S et al. *JAMA Network Open.*

2002-2012	68 (52.3)
2013-2017	62 (47.7)

Abbreviations: 3DCRT, three-dimensional conformal radiation therapy; IMRT, intensity-modulated radiation therapy.

© 2023 Zhou S et al. *JAMA Network Open.*

eTable 2. Association of Siglec-15 and PD-L1 Expression With Patient Clinicopathological Characteristics in the Primary Cohort

Characteristic	Siglec-15 Expression		*P*-value	PD-L1 Expression		*P*-value
	Positivity	Negativity		Positivity	Negativity	
Age (years)			0.77			0.27
<56	32 (45.7%)	29 (48.3%)		36 (51.4%)	25 (41.7%)	
≥56	38 (54.3%)	31 (51.7%)		34 (48.6%)	35 (58.3%)	
Sex			0.59			0.39
Male	57 (81.4%)	51 (85.0%)		60 (85.7%)	48 (80.0%)	
Female	13 (18.6%)	9 (15.0%)		10 (14.3%)	12 (20.0%)	
Smoking history			0.58			0.33
Yes	47 (67.1%)	43 (71.7%)		51 (72.9%)	39 (65.0%)	
No	23 (32.9%)	17 (28.3%)		19 (27.1%)	21 (35.0%)	
Alcohol history			0.45			0.27
Yes	29 (41.4%)	21 (35.0%)		30 (42.9%)	20 (33.3%)	
No	41 (58.6%)	39 (65.0%)		40 (57.1%)	40 (66.7%)	
Performance status			0.53			0.25
0	40 (57.1%)	31 (51.7%)		35 (50.0%)	36 (60.0%)	
1–2	30 (42.9%)	29 (48.3%)		35 (50.0%)	24 (40.0%)	
Weight loss			0.38			0.78
<10%	62 (88.6%)	56 (93.3%)		64 (91.4%)	54 (90.0%)	
≥10%	8 (11.4%)	4 (6.7%)		6 (8.6%)	6 (10.0%)	
Histologic grade			0.19			0.62
Gx/1/2	49 (70.0%)	48 (80.0%)		51 (72.9%)	46 (76.7%)	
G3	21 (30.0%)	12 (20.0%)		19 (27.1%)	14 (23.3%)	
Tumor location			0.03			0.62
Upper/middle	47 (67.1%)	50 (83.3%)		51 (72.9%)	46 (76.7%)	

© 2023 Zhou S et al. *JAMA Network Open.*

Distal	23 (32.9%)	10 (16.7%)		19 (27.1%)	14 (23.3%)	
Primary tumor length			0.71			0.08
≤5 cm	35 (50.0%)	28 (46.7%)		29 (41.4%)	34 (56.7%)	
>5 cm	35 (50.0%)	32 (53.3%)		41 (58.6%)	26 (43.3%)	
Clinical T stage			0.38			0.38
T1-2	11 (15.7%)	13 (21.7%)		11 (15.7%)	13 (21.7%)	
T3-4	59 (84.3%)	47 (78.3%)		59 (84.3%)	47 (78.3%)	
Clinical N stage			0.80			0.69
N0	5 (7.1%)	5 (8.3%)		6 (8.6%)	4 (6.7%)	
N1-3	65 (93.9%)	55 (91.7%)		64 (91.4%)	56 (93.3%)	
Treatment era			0.20			0.40
2002-2012	33 (47.1)	35 (58.3)		39 (55.7)	29 (48.3)	
2013-2017	37 (52.9)	25 (41.7)		31 (44.3)	31 (51.7)	

Abbreviations: Siglec-15, sialic acid-binding immunoglobulin-like lectin 15; PD-L1, programmed cell death-ligand 1.

© 2023 Zhou S et al. *JAMA Network Open.*

eTable 3. Univariate and Multivariate Analyses for Variables Associated With Pathologic Complete Response in the Primary Cohort

Variable	Univariate		Multivariate	
	Odds ratio (95% CI)	*P*-value	Odds ratio (95% CI)	*P*-value
Age (<56 vs. ≥56)	0.76 (0.38–1.52)	0.43		
Sex (female vs. male)	1.62 (0.64–4.07)	0.31		
Smoking history (yes vs. no)	0.63 (0.30–1.34)	0.23		
Alcohol history (yes vs. no)	0.84 (0.41–1.72)	0.64		
Performance status (0 vs. 1–2)	0.71 (0.36–1.43)	0.34		
Weight loss (<10% vs. ≥10%)	0.37 (0.11–1.29)	0.12		
Histologic grade (Gx/1/2 vs. G3)	0.96 (0.43–2.11)	0.91		
Tumor location (upper/middle vs. distal)	1.13 (0.51–2.50)	0.77		
Primary tumor length (≤5 vs. >5 cm)	0.99 (0.49–1.97)	0.97		
Clinical T stage (T1-2 vs. T3-4)	1.06 (0.44–2.59)	0.89		
Clinical N stage (N0 vs. N1-3)	0.82 (0.22–3.04)	0.76		
Chemotherapy regimen[a] (1 vs. 2/3)	1.63 (0.78–3.40)	0.20		
Radiation dose (≤40 vs. >40 Gy)	0.81 (0.37–1.79)	0.61		
Radiotherapy modality (3DCRT vs. IMRT)	1.03 (0.47–2.26)	0.95		
Treatment era (2002-2012 vs. 2013-2017)	0.85 (1.03–4.23)	0.64		
Siglec-15 (positivity vs. negativity)	2.08 (0.42–1.69)	0.04	2.08 (1.03–4.23)	0.04
PD-L1 (positivity vs. negativity)	1.42 (0.71–2.85)	0.33		

Abbreviations: CI, confidence interval; 3DCRT, three-dimensional conformal radiation therapy; IMRT, intensity-modulated radiation therapy; Siglec-15, sialic acid-binding immunoglobulin-like lectin 15; PD-L1, programmed cell death-ligand 1.

[a]Chemotherapy regimen: 1, cisplatin/vinorelbine; 2, cisplatin/fluorouracil; 3, cisplatin/taxane.

© 2023 Zhou S et al. *JAMA Network Open.*

eTable 4. Multicollinearity Test for Variables in the Multivariate Regression Model

Variable	*P*-value	Tolerance	Variance inflation factor
Age (<56 vs. ≥56)	0.02	0.96	1.04
Sex (female vs. male)	0.07	0.94	1.07
Primary tumor length (≤5 vs. >5 cm)	0.45	0.91	1.10
Pathologic response (pCR vs. non-pCR)	0.04	0.95	1.05
Siglec-15 (positivity vs. negativity)	0.003	0.95	1.05
PD-L1 (positivity vs. negativity)	0.001	0.94	1.06

© 2023 Zhou S et al. *JAMA Network Open.*

eTable 5. Patient Characteristics in the Independent Validation Cohort

Characteristic	Total (n = 55), %
Age (years)	
<56	17 (30.9)
≥56	38 (69.1)
Sex	
Male	43 (78.2)
Female	12 (21.8)
Smoking history	
Yes	27 (49.1)
No	28 (50.9)
Alcohol history	
Yes	23 (41.8)
No	32 (58.2)
Performance status	
0	42 (76.4)
1–2	13 (23.6)
Weight loss	
<10%	49 (89.1)
≥10%	6 (10.9)
Histologic grade	
Gx/1/2	40 (72.7)
G3	15 (27.3)
Tumor location	
Upper	7 (12.7)
Middle	33 (60.0)

© 2023 Zhou S et al. *JAMA Network Open.*

Distal	15 (27.3)
Primary tumor length	
≤5 cm	25 (45.5)
>5 cm	30 (54.5)
Clinical T stage	
T1-2	16 (29.1)
T3-4	39 (70.9)
Clinical N stage	
N0	1 (1.8)
N1-3	54 (98.2)
Clinical TNM stage	
I/II	4 (7.3)
III	51 (92.7)
Chemotherapy regimen	
Cisplatin/vinorelbine	22 (40.0)
Cisplatin/fluorouracil	6 (10.9)
Cisplatin/taxane	27 (49.1)
Radiation dose (Gy)	
Median (range)	44.0 (44.0–45.0)
Radiotherapy modality	
IMRT	55 (100.0)
Surgical procedure	
Ivor-Lewis esophagectomy	55 (100.0)

Abbreviations: 3DCRT, three-dimensional conformal radiation therapy; IMRT, intensity-modulated radiation therapy.

© 2023 Zhou S et al. *JAMA Network Open.*
