# Supplementary material for: Association of Sialic Acid–Binding Immunoglobulin-Like Lectin 15 With Phenotypes in Esophageal Squamous Cell Carcinoma in the Setting of Neoadjuvant Chemoradiotherapy
Source: JAMA Netw Open. 2023 Jan 17;6(1):e2250965. doi: 10.1001/jamanetworkopen.2022.50965 (PMC9856737; doi:10.1001/jamanetworkopen.2022.50965)
Supplement: Supplement 2. — Data Sharing Statement [file jamanetwopen-e2250965-s002.pdf]

Data Sharing Statement

Zhou. Association of Sialic Acid-Binding Immunoglobulin-Like Lectin 15 With Phenotypes in Esophageal Squamous Cell Carcinoma in the Setting of Neoadjuvant Chemoradiotherapy. *JAMA Netw Open*. Published January 17, 2023. doi:10.1001/jamanetworkopen.2022.50965

Data
Data available: No
